# Magnesium and memantine do not inhibit nociceptive neuronal activity in the trigeminocervical complex of the rat

**DOI:** 10.1186/1129-2377-14-S1-P71

**Published:** 2013-02-21

**Authors:** J Hoffmann, JW Park, RJ Storer, PJ Goadsby

**Affiliations:** 1 Headache Group - Department of Neurology, University of California San Francisco, USA

## Introduction

Experimental studies with NMDA receptor antagonists, such as magnesium and memantine, have demonstrated the ability of these substances to inhibit nociceptive trigeminal neurotransmission in electrophysiological studies. Despite these promising experimental results clinical trials results have been less than clear. To investigate further this contrast using the open channel blockers, magnesium and memantine, we studied their effects in a model of nociceptive trigeminovascular activation.

## Methods

Sprague-Dawley rats were anesthetized with pentobarbital (60 mgkg-1) and cannulated for physiological monitoring, maintenance of further anesthesia and drug administration. Anesthesia was maintained with intravenous propofol (20-25 mgkg-1h-1). A cranial window was prepared over the middle meningeal artery (MMA) and a bipolar electrode was placed on the dura mater above the MMA for electrical stimulation. For recording of neuronal activity a tungsten electrode was introduced in the trigeminocervical complex (TCC). Experimental groups received either intravenously administered memantine (10 mgkg-1), magnesium (100 mgkg-1) or vehicle.

## Results

Magnesium and memantine did not have a significant inhibitory effect on neuronal activity in the TCC. However, blood pressure was significantly reduced after memantine (31 ± 5 %, p < 0.05) or magnesium (49 ± 11 %, p < 0.05) administration when compared to baseline.

## Conclusion

The results indicate that the known inhibitory effect of magnesium and memantine after microiontophoretic application in the TCC could not be reproduced with intravenous administration. This might be a result of the low drug concentration that can be achieved with this route of administration at the relevant site of action. A further increase in dosage is not feasible since the used dosage already led to significant reductions in arterial blood pressure. The results support the clinical observations and provide a possible explanation for the lack of consistent efficacy of these drugs for the treatment of migraine.
